# COVID-19 and Acute Ischemic Stroke Mortality and Clinical Outcomes among Hospitalized Patients in the United States: Insight from National Inpatient Sample

**DOI:** 10.3390/jcm12041340

**Published:** 2023-02-08

**Authors:** Monique G. Davis, Karthik Gangu, Sajid Suriya, Babu Sriram Maringanti, Prabal Chourasia, Aniesh Bobba, Alok Tripathi, Sindhu Reddy Avula, Rahul Shekhar, Abu Baker Sheikh

**Affiliations:** 1 Department of Internal Medicine, University of New Mexico Health Sciences Center, Albuquerque, NM 87106, USA; 2 Department of Internal Medicine, University of Kansas Medical Center, Kansas City, KS 66160, USA; 3 Department of Neurology, University of New Mexico Health Sciences Center, Albuquerque, NM 87106, USA; 4 Department of Hospital Medicine, Mary Washington Hospital, Fredericksburg, VA 22401, USA; 5 Department of Medicine, John H Stronger Hospital, Cook County, Chicago, IL 60612, USA; 6 Department of Interventional Cardiology, Division of Cardiology, The University of Kansas Health System St. Francis Campus, Kansas City, KS 66606, USA

**Keywords:** COVID-19, ischemic, stroke, cerebral vascular accident, mortality, prevalence, complications, United States, National Inpatient Sample

## Abstract

Coronavirus-19, primarily a respiratory virus, also affects the nervous system. Acute ischemic stroke (AIS) is a well-known complication among COVID-19 infections, but large-scale studies evaluating AIS outcomes related to COVID-19 infection remain limited. We used the National Inpatient Sample database to compare acute ischemic stroke patients with and without COVID-19. A total of 329,240 patients were included in the study: acute ischemic stroke with COVID-19 (*n* = 6665, 2.0%) and acute ischemic stroke without COVID-19 (*n* = 322,575, 98.0%). The primary outcome was in-hospital mortality. Secondary outcomes included mechanical ventilation, vasopressor use, mechanical thrombectomy, thrombolysis, seizure, acute venous thromboembolism, acute myocardial infarction, cardiac arrest, septic shock, acute kidney injury requiring hemodialysis, length of stay, mean total hospitalization charge, and disposition. Acute ischemic stroke patients who were COVID-19-positive had significantly increased in-hospital mortality compared to acute ischemic stroke patients without COVID-19 (16.9% vs. 4.1%, aOR: 2.5 [95% CI 1.7–3.6], *p* < 0.001). This cohort also had significantly increased mechanical ventilation use, acute venous thromboembolism, acute myocardial infarction, cardiac arrest, septic shock, acute kidney injury, length of stay, and mean total hospitalization charge. Further research regarding vaccination and therapies will be vital in reducing worse outcomes in patients with acute ischemic stroke and COVID-19.

## 1. Introduction

The Severe Acute Respiratory Virus Syndrome Coronavirus 2 (SARS-CoV-2), first detected in Wuhan, China, in December 2019, has caused a worldwide pandemic known as Coronavirus-19 (COVID-19) [1,2]. Despite primarily being a respiratory virus, it also affects multiple organ systems, including the nervous system [3]. Neurological complications have been documented in approximately 36.4% of COVID-19 patients, and roughly 28.2% had severe central nervous system (CNS) injuries, contributing to worse morbidity and mortality [4]. Known neurological complications include acute ischemic stroke (AIS), neuropsychiatric symptoms, encephalopathy, seizures, myopathies, and polyradiculoneuropathies [5]. COVID-19 infection is associated with worse outcomes in AIS patients [6,7]; however, large-scale studies evaluating AIS outcomes with COVID-19 infection are limited. 

The association of AIS with COVID-19 infection is well-established by multiple studies due to the increased reports of this condition during the pandemic [3,8,9,10]. Ischemic stroke refers to a reduction in blood flow to part or all of the brain resulting in tissue damage and can be characterized as an acute, subacute, or chronic process [11]. Ischemic stroke subtypes can also be categorized using the Trial of ORG 10172 in Acute Stroke Treatment (TOAST) classification based on the underlying etiology (i.e., large artery atherosclerosis or large vessel occlusion [LVO]; cardioembolism; small vessel occlusion; stroke of other, unusual, determined etiology; and stroke of undetermined etiology) [11]. LVOs constitute the greatest proportion (37.3%) of AIS, with the middle cerebral artery as the most common (49.6%) culprit [11]. Globally, there is a slight difference between sex and incidence of AIS, and this slight predominance in females between countries is due to AIS being more common among older ages and women dominating in senescence [5,12]. Its signs and symptoms are highly variable and include sudden unilateral face and extremity weakness or focal deficits, slurred speech, confusion, vision changes, and death [12]. AIS is associated with elevated biomarkers such as prothrombin time (PT), activated partial thromboplastin time (aPTT), c-reactive protein (CRP), D-dimer, fibrinogen, lactate dehydrogenase (LDH), and ferritin [3]. Clinical presentation and neuroimaging (computed tomography angiography [CTA] or CT perfusion studies) remain the mainstays for the evaluation and diagnosis [10].

The pathophysiology of AIS in COVID-19 infection remains unclear and debatable [3,8]. It is hypothesized to involve a prothrombotic state induced by the hyperinflammatory response from the underlying COVID-19 infection, which leads to elevated antiphospholipid antibodies, von Willebrand factor, factor VIII, fibrinogen, and D-dimer markers [3]. Another mechanism is believed to be due to the binding of the angiotensin-converting enzyme (ACE) 2 receptor and the resultant downregulation of blood pressure [4]. Additionally, cryptogenic causes are thought to be very common in this population, including endothelial dysfunction and cardiomyopathy after a viral or respiratory illness, resulting in a heightened risk of stroke [5,12,13]. 

Our study’s objective is to assess outcomes between AIS patients with and without COVID-19 infection, by utilizing data from the National Inpatient Sample (NIS). The primary outcome was in-hospital mortality. Secondary outcomes included mechanical ventilation, vasopressor use, mechanical thrombectomy, thrombolysis, seizure, acute venous thromboembolism (VTE), acute myocardial infarction (MI), cardiac arrest, septic shock, acute kidney injury (AKI), AKI requiring hemodialysis (HD), length of stay (LOS), mean total hospitalization charge, and disposition.

## 2. Materials and Methods

This retrospective study utilized the NIS Healthcare Cost Utilization Project [HCUP], sponsored by the Agency for Healthcare Research and Quality [AHRQ] database, which is an all-payer database that approximates a 20% stratified sample of discharges from US community hospitals [14]. In this analysis, we used the 2020 National Inpatient Sample (NIS) data set, which included hospitalizations from 1 January 2020 to 31 December 2020, and was made available to the public in October 2022. The NIS database contains a de-identified collection of billing and diagnostic codes from participating hospitals. The NIS dataset does not directly involve ‘human subjects’ (consistent with federal regulations and guidance [15]) and is exempt from institutional review board approval.

### 2.1. Inclusion and Exclusion Criteria

All patients of age ≥ 18 years, admitted to the hospital with acute ischemic stroke and COVID-19 infection, were included in the study. We used ICD-10 clinical modification (CM) codes to retrieve patient samples and comorbid conditions, and ICD-10 procedure codes were used to identify in-hospital procedures. A detailed code summary is provided in Supplementary Table S1. Patient’s age < 18 years, inter-hospital transfers, missing NIH stroke scale (NIHSS) on admission, contrast allergy, and hemorrhagic stroke were all excluded from the study (Supplemental Figure S1).

### 2.2. Covariates

The NIS data sample contains data regarding in-hospital outcomes, procedures, and other discharge-related information. Variables were divided into patient level, hospital level, and illness severity.

- Patient-level: age, race, sex, comorbidities, insurance status, income in the patient’s zip code, disposition;
- Hospital level: location, teaching status, bed size, region;
- Illness severity: length of stay (LOS), mortality, hospitalization cost, Elixhauser comorbidity score, in-hospital complications, mechanical ventilation, vasopressor use, cardiac arrest, acute MI, acute VTE, seizures, septic shock, and NIHSS.

### 2.3. Study Outcomes

The primary outcome assessed was in-hospital mortality. Secondary outcomes were (a) intubation rate and vasopressor use; (b) acute MI, AKI and AKI requiring HD, andVTE; (c) tPA and mechanical thrombectomy use; (d) septic shock and cardiac arrest; (e) length of stay; (f) financial burden on healthcare; and (g) resource utilization.

### 2.4. Statistical Methods

STATA 17 (StataCorp LLC, College Station, TX, USA) was utilized for statistical analysis [16]. The unweighted sample was 6.47 million observations, and the weighted sample was around 32.3 million discharges for the year 2020. Patients who were admitted with acute ischemic stroke were retrieved with ICD-10 CM codes, and this group was further divided based on COVID status. The chi-square test was used to compare categorical variables, and linear regression was used for continuous variables. For the primary outcome, univariate logistic regression was used to calculate the unadjusted odds ratio for variables of interest, and *p* values of <0.2 on univariate logistic regression were used to build a multivariate logistic regression model to adjust for potential confounders. A multivariate linear regression model was used for continuous variables (length of stay and total hospital charge). A two-tailed *p*-value of 0.05 was considered significant. We conducted a secondary analysis with propensity matching to confirm the results obtained by traditional multivariate analysis. Baseline demographics (Age, race, sex, income status, and insurance status) were matched using a 1:1 nearest neighbor propensity score with 0.05 caliper width n matched cohort, and a secondary multivariate regression model was built as described above (Supplementary Figure S2). All analysis was performed using Stata software version 17.0 (Stata Corporation, College Station, TX, USA). *p* values of less than 0.05 were considered statistically significant.

## 3. Results

### 3.1. Demographics and Baseline Comorbidities

In our study population, a total of 329,240 hospitalized acute ischemic stroke patients, out of which 6665 (2.0%) were diagnosed with AIS with a concomitant COVID-19 infection, between 1 January 2020 to 31 December 2020, were included. AIS patients who were COVID-19-positive were more often females (43.4% vs. 48.3%, *p* < 0.001) and had a greater proportion of African Americans (21.8% vs. 17.6%, *p* < 0.001), Hispanics (18.8% vs. 8.0%, *p* < 0.001), Asians (3.6% vs. 3.2%, *p* < 0.001), and Native Americans (0.8% vs. 0.4%, *p* < 0.001). AIS with COVID-19 infection was significantly more prevalent among patients aged 18 to 29 years (0.68% vs. 0.63%, *p* = 0.009), 30 to 49 years (10.6% vs. 8.2%, *p* = 0.009), and 50 to 69 years (40.2% vs. 39.3%, *p* = 0.009), with mean ages of 69.5 ± 15.3 years for females and 66.2 ± 13.0 years for males. This cohort was also more likely to have a median household income below 50,000 USD (34.2% vs. 29.8%, *p* = 0.03) compared to AIS patients without COVID-19 (Table 1).

Patients with AIS and COVID-19 were significantly more obese (19.1% vs. 16.9%, *p* = 0.03) and more likely to be diabetic (46.0% vs. 38.6%, *p* < 0.001). Patients with AIS alone had significantly higher rates of smoking (40.8% vs. 25.7%, *p* < 0.001), alcohol abuse (5.0% vs. 3.8%, *p* = 0.04), and coronary artery disease (CAD) (25.6% vs. 22.6%, *p* = 0.01). There was no significant difference in regard to congestive heart failure (CHF), hypertension, cardiac arrhythmias, chronic pulmonary disease, or chronic kidney disease (CKD) between the two cohorts (Table 1). 

According to the National Institutes of Health Stroke Scale (NIHSS), AIS patients with concurrent COVID-19 infection had significantly higher scores from 10 to 19 (22.7% vs. 15.2%, *p* < 0.001), 20 to 29 (13.0% vs. 7.2%, *p* < 0.001), and 30 to 39 (4.0% vs. 1.3%, *p* < 0.001) than patients with AIS only (Table 1). 

There was some variability in the geographic distribution of patients. Most patients in both cohorts were seen at urban teaching hospitals (83.9% vs. 79.6%, respectively) and had higher proportions of Medicare beneficiaries (60.5% vs. 64.0%, respectively, *p* = 0.02). The baseline characteristics of the study cohorts are outlined in Table 1. The baseline characteristics of the matched cohort after the propensity matching are provided in Supplementary Table S2.

### 3.2. In-Hospital Mortality

Multivariate logistic regression was performed and adjusted for patient age, sex, race, discharge quarter, Elixhauser comorbidity index, teaching status, bed size, and insurance status (Table 2). In-hospital mortality (*n* = 13,990, 4.2%) was significantly higher in patients with AIS and COVID-19 infection compared to those who were COVID-19-negative (16.8% vs. 4.0%, adjusted OR [aOR]: 3.3 [95% CI 2.7–4.1], *p* < 0.001) (Table 2). In COVID-19 patients without AIS, mortality was 13.1% (*n* = 214,040, sample size = 1,633,040).

Mortality was further divided by evaluating gender, race, and age. Mortality in AIS patients with COVID-19 was significantly higher among males (61.6% vs. 48.6%, *p* = 0.003), African Americans (17.4% vs. 12.0%, *p* < 0.001), Hispanics (21.9% vs. 7.3%, *p* < 0.001), Asians (5.4% vs. 3.6%, *p* < 0.001), and Native Americans (1.3% vs. 0.5%, *p* < 0.001) as compared to patients with AIS alone. Mortality for AIS patients without COVID-19 was significantly predominant among females (51.4% vs. 38.4%, *p* = 0.003) and Caucasians (73.3% vs. 49.1%, *p* < 0.001) when compared to AIS patients with COVID-19 (Table 3). 

Propensity matching was performed regarding patient age, sex, race, income, insurance status, and Elixhauser (Supplementary Table S2). After PSM, there were a total of 6290 patients in each group (with COVID-19 and without COVID-19) (Supplementary Table S2). The in-hospital mortality (*n* = 1315) remained significantly higher in AIS patients with COVID-19 compared to those without COVID-19 (16.9% vs. 4.1%, aOR: 2.5 [95% CI 1.7–3.6], *p* < 0.001) (Table 4). 

### 3.3. In-Hospital Complications

Patients who presented with AIS with COVID-19 required more mechanical ventilation (20.5% vs. 7.1%, aOR: 1.9 [95% CI 1.6–2.3], *p* < 0.001) and mechanical thrombectomy (8.9% vs. 7.5%, aOR: 0.8 [95% CI 0.6–1.0], *p* = 0.02). These patients also experienced significantly higher rates of acute venous thromboembolism (7.8% vs. 2.6%, aOR: 2.5 [95% CI 2.0–3.0], *p* < 0.001), acute myocardial infarction (8.3% vs. 4.2%, aOR: 1.5 [95% CI 1.2–1.9], *p* < 0.001), cardiac arrest (3.4% vs. 0.7%, aOR: 2.5 [95% CI 1.7–3.7], *p* < 0.001), septic shock (5.0% vs. 0.9%, aOR: 2.6 [95% CI 1.9–3.6], *p* < 0.001), acute kidney injury (27.8% vs. 15.3%, aOR: 1.6 [95% CI 1.4–1.9], *p* < 0.001), and acute kidney injury requiring hemodialysis (2.1% vs. 0.4%, aOR: 1.8 [95% CI 1.1–2.9], *p* = 0.01). This cohort did not require significant vasopressor use (3.2% vs. 1.0%, aOR: 1.2 [95% 0.8–1.8], *p* = 0.44) and thrombolysis (12.9% vs. 12.6%, aOR: 0.9 [95% CI 0.8–1.1], *p* = 0.50), nor did they experience significant rates of seizure (3.9% vs. 4.1%, aOR: 0.8 [95% CI 0.6–1.0], *p* = 0.08) (Table 2).

After PSM, patients with AIS and COVID-19 continued to require more mechanical ventilation (20.2% vs. 6.2%, aOR: 2.1 [95% CI 1.6–2.8], *p* < 0.001) but no longer required significant mechanical thrombectomy use (8.8% vs. 6.8%, aOR: 0.8 [95% CI 0.6–1.2], *p* = 0.23). Higher rates of acute venous thromboembolism (8.1% vs. 2.5%, aOR: 2.6 [95% CI 1.7–3.9], *p* < 0.001), acute myocardial infarction (8.5% vs. 3.7%, aOR: 1.7 [95% CI 1.2–2.6], *p* = 0.008), cardiac arrest (3.1% vs. 0.4%, aOR: 4.2 [95% CI 1.5–11.4], *p* = 0.005), septic shock (4.8% vs. 0.7%, aOR: 3.3 [95% CI 1.5–7.4], *p* = 0.003), and acute kidney injury (28.1% vs. 14.3%, aOR: 1.8 [95% CI 1.5–2.3], *p* < 0.001) were consistently observed in this cohort. This group no longer had significant rates of acute kidney injury requiring hemodialysis (2.0% vs. 0.5%, aOR: 1.9 [95% CI 0.7–5.1], *p* = 0.22). Patients with AIS and COVID–19 infection did not have significant vasopressor use (3.1% vs. 1.0%, aOR: 1.4 [95% 0.7–2.6], *p* = 0.35), thrombolysis use (12.6% vs. 10.8%, aOR: 1.1 [95% CI 0.9–1.5], *p* = 0.37), or seizure rates (3.8% vs. 3.7%, aOR: 1.0 [95% CI 0.6–1.6], *p* = 0.93) (Table 4).

### 3.4. In-Hospital Quality Measures and Disposition

Patients with COVID-19-associated AIS had an increased mean length of stay (10.2 days vs. 5.4 days, adjusted length of stay 3.2 days higher, *p* < 0.001) in comparison to AIS patients without COVID-19 infection. Patients with AIS and COVID-19 had a higher mean total hospitalization charge (145,497 USD vs. 88,320 USD, adjusted total charge 25,814 USD higher, *p* < 0.001). Of those who survived, patients with COVID-19-related AIS had more discharges to skilled nursing facilities (SNF), long-term acute care (LTAC) facilities, and nursing homes (48.3% vs. 40.8%, *p* < 0.001), as well as more discharges for home health (20.3% vs. 19.7%, *p* < 0.001) compared to those with AIS alone (Table 2).

After PSM, AIS patients who were COVID-19-positive continued to have an increased mean length of stay (10.2 days vs. 5.7 days, adjusted length of stay 2.8 days higher, *p* < 0.001) and a higher mean total hospitalization charge (145,287 USD vs. 84,419 USD, adjusted total charge 31,400 USD higher, *p* < 0.001) than COVID-19-negative AIS patients (Table 4).

## 4. Discussion

In this retrospective analysis, we identified 329,240 hospitalized patients diagnosed with acute ischemic stroke between 1 January 2020 and 31 December 2020, out of which 6665 (0.4%) were diagnosed with AIS and COVID-19 infection, with a total of 1,659,040 COVID-19 hospitalizations. Our study included 322,575 AIS patients without COVID-19 during the same duration. Major findings of our study include: (1) AIS patients with COVID-19 had significantly increased in-hospital mortality compared to AIS patients who were COVID-19-negative. (2) Patients in the AIS with COVID-19 cohort required more mechanical ventilation and had significantly higher rates of acute VTE, acute MI, cardiac arrest, septic shock, and AKI. (3) AIS and COVID-19 patients had significantly higher NIHSS scores. (4) In-hospital mortality was higher among males, African Americans, Hispanics, Asians, and Native Americans with COVID-19-positive AIS than among females and Caucasians with COVID-19-negative AIS. 

The prevalence of AIS with COVID-19 was reported as 0.2–2.7% in previous publications, which is consistent with our prevalence data of 0.4% [3,8,9,17]. A recent systematic review and meta-analysis by Nagraj et al. reviewed the incidence of stroke in randomized trials for COVID-19 therapeutics and included studies until 30 July 2021 [18]. This systematic review included 38,732 patients from inpatient and outpatient settings (65 patients with stroke) from 77 randomized studies, and all reported stroke events happened in the inpatient setting with an overall incidence of 0.18%. In contrast, our study included 329,240 inpatients (6665 patients with stroke) and found worse outcomes in patients with AIS and COVID-19 infection, with an overall incidence of 0.4%. They also concluded that there is a possibility of an association between the severity of the COVID-19 infection with the occurrence of stroke. Compared to prior studies, which have included smaller sample sizes [6,18], our study compares AIS outcomes in COVID-19 patients based on one of the largest cohorts of COVID-19 patients hospitalized in the United States in 2020. To our knowledge, our study is the largest study looking at the inpatient outcomes of patients hospitalized with AIS and COVID-19.

In the literature, patients with AIS who were COVID-19-positive were predominantly males, ranging from 58% to 62.3% [3,8]. Our study’s results suggested that women were the most affected gender; however, when a mortality breakdown was performed, males were more likely than females to die from concomitant AIS and COVID-19. In AIS patients who were COVID-19-negative, females had the greatest predisposition toward mortality, correlating with the literature [19]. Our study contains a larger sample size, likely impacting the difference regarding which gender is most affected. We did not find any large studies investigating gender as a risk factor for mortality in COVID-19-positive AIS. Women have a slight predominance for ischemic stroke among non-COVID-19 cases, due to having a longer lifespan than males and an increased risk of AIS in senescence; however, men have become more afflicted in the setting of COVID-19 [8,19]. This increased occurrence of COVID-19 in men is consistent with the gender distribution observed in COVID-19 complications [20]. Men are more likely than women to succumb to physical stress (i.e., inflammation and infection) [8] due to the pro-inflammatory effects of testosterone, contrary to estrogen, which is protective and reduces inflammation [21]. 

In AIS alone, less than half of the data from the United States and the Canadian AIS randomized controlled trials include details about ethnicity and race [22]. In AIS with COVID-19 infection, racial disparities are underreported as well; however, one retrospective study found that African American patients with COVID-19 had a significantly higher likelihood of stroke (50%, *p* = 0.03) than their non-African American counterparts [7,23]. Another study found that Native Americans had a higher prevalence of conditions associated with COVID-19, such as cerebrovascular accidents, due to a higher predilection toward vascular risk factors (i.e., hypertension, atrial fibrillation, CAD, obesity, smoking, and alcohol abuse [24]. Our study suggests African Americans, Hispanics, Asians, and Native Americans, in descending order, are more predisposed to concurrent AIS and COVID-19. Furthermore, when mortality was divided, our results showed that these same races were also risk factors for mortality in this cohort, whereas Caucasians were more susceptible to mortality in COVID-19-negative AIS patients. We did not find any large studies investigating race as a specific risk factor for mortality, and these studies would be beneficial. These racial disparities can best be explained by socio-economic factors; however, definitive conclusions on causality for these findings cannot be drawn [25,26,27,28].

With respect to age, our patients with COVID-19-related AIS had increasing susceptibilities to develop this condition when aged between 18 and 69 years, with mean ages of 69.5 ± 15.3 and 66.2 ± 13.0 years for females and males, respectively. Other published data were similar to these findings, with a documented age range from 48.1 to 75.7 years and a mean age of 63.4 ± 13.1 years [3,8]. Moreover, AIS in the setting of COVID-19 affects more young patients than during pre-pandemic times [29]. Although our study did not find that age was a predictor of mortality, the literature shows that poor functionality and mortality are higher with increasing age in this population [29]. Older patients, 70 years and above, with only COVID-19 infection, have been shown to have mortality between 10.9% and 26.6%, possibly due to many reasons, including aging immunity, numerous comorbidities, and congregate housing [30]. Age in AIS with COVID-19 infection could benefit from further studies as the pandemic continues. 

Per our data, patients with AIS and COVID-19 were significantly more obese and more likely to have diabetes mellitus, which was supported by Luo et al., among other studies, in which the prevalence of diabetes in this group ranged from 20% to 65% with a pooled prevalence of 40% [3,8]. Our cohort with AIS alone was significantly susceptible to smoking, alcohol abuse, and CAD; however, it is unclear why these risk factors would not contribute to a predisposition to COVID-19 in our study. Prospective studies would be helpful for clarifying this discussion. Although we did not have data pertaining to hyperlipidemia, and there was no significant difference found for hypertension between our cohorts, common cerebrovascular comorbidities such as hypertension and hyperlipidemia, along with diabetes, were most frequently observed throughout the literature in patients with concomitant AIS and COVID-19 infection [3,8]. Other reports indicate that 22% to 24% of the COVID-19-associated AIS population did not have any known medical history or substance use, and approximately 44% of these patients presented with at least four vascular risk factors, which likely contributed to case severity [31].

In our study, patients with both AIS and COVID-19 had significantly higher in-hospital mortality at 16.9%, which was lower than the mortality of 20% to 63.6% documented in the literature yet higher than those with COVID-19 without AIS at 13.1% [5,9,10]. When further mortality analysis was performed, which evaluated gender, race, and age, it was found that gender and race have a significant role in mortality outcomes in COVID-19-positive AIS patients, as discussed above. This high mortality can be explained by the additive effect of the COVID-19 infection. COVID-19 infection leading to a cytokine storm, excessive systemic inflammatory response, coagulation dysfunction, oxidative stress, direct neurological injury, and hypoxia-induced multiorgan failure all contributed to an increase in mortality, along with the lack of effective and available therapies and vaccinations for COVID-19 during the early phase of the pandemic and the characteristics and comorbidities discussed above. Additionally, social consequences of the pandemic (i.e., the overwhelmed healthcare system or fear of contracting COVID-19 if one sought healthcare) led patients to delay care, which may have caused the progression of a mild stroke into the moderate to severe presentations observed on admission [32,33]. It is important to recognize and treat AIS early and aggressively, in order to improve morbidity and mortality [33].

Our cohort of COVID-19-associated AIS patients had significantly increased mechanical ventilation requirements, acute VTE, acute MI, cardiac arrest, septic shock, and AKI compared to the non-COVID-19 AIS cohort. These results were consistent with the literature [5,8,34]. Ganesh et al. noted that up to 38% of AIS patients with high NIHSS scores undergo intubation, and this value is presumed to be even higher when combined with the complexity of COVID-19 [5]. Patients become additionally vulnerable to AKI, and thus, mortality increases when contrasted neuroimaging is used as part of the AIS workup [5]. Regarding elevated prothrombotic markers, studies show approximately 41.7% of COVID-19-associated AIS patients test positive for antiphospholipid antibodies, and 72.6% have elevated D-dimer levels [3]. That study, among others, suggested the severity of stroke presentation and worsened outcomes correlated with prothrombotic and inflammatory markers [3,8]. Moreover, worse outcomes can be explained by the reliance on biomarkers and clinical suspicion (regardless of neurological history) when imaging was not readily available for confirmatory diagnosis or when the use of standard modalities was delayed due to the early pandemic isolation effect [32,33]. Our study is limited due to the exclusion of lab values and imaging, as NIS diagnoses are based on ICD-10 codes. Despite higher NIHSS scores and therefore increased stroke severity in this population, our COVID-19-related AIS cohort did not require significant use of mechanical thrombectomy or thrombolysis. Irrespective of our results, there is accumulating evidence of negative outcomes (over a 9-fold increase in mortality) in AIS patients with COVID-19 who receive mechanical thrombectomy regardless of the timing and success of the intervention, necessitating risk-benefit discussions with these patients [35]. 

With respect to NIHSS scores, our cohort of AIS patients with concurrent COVID-19 infection had significantly higher scores. This correlated with the literature in that patients with AIS with COVID-19 had moderate-to-severe stroke severity on admission compared to non-COVID-19 cohorts, which may explain the poor outcomes, as analyzed above [6,36]. In a systematic review and meta-summary by Tan et al., the mean NIHSS score was 19 ± 8, consistent with LVOs observed in 40.9% of AIS with COVID-19 patients [3], and was also proportional to increased mortality [9]. A limitation of our study was the exclusion of stroke subtypes within our cohort data. 

In our study, the co-diagnosis of AIS and COVID-19 resulted in a significantly increased length of stay, an increased mean total hospitalization charge, and more discharges to a facility or home services for continued care than non-COVID-19 AIS patients. These results were predictable given that patients with AIS and COVID-19 had significant complications and required higher levels of support, as discussed above. 

AIS treatment in the general, non-COVID-19 population primarily includes blood pressure control, anticoagulation, long-term dual antiplatelet agents and statin therapy, potential thrombolysis using recombinant tissue plasminogen activator (rt-PA; i.e., alteplase) and mechanical thrombectomy depending on the case and if the patient is within the therapeutic window of 3 to 4.5 h or within 6 h, respectively [3,12,37,38]. The mainstay of management for COVID-19-associated AIS is the aforementioned stroke treatment in combination with supportive care and treatment of the underlying COVID-19 infection [10]. COVID-19 patients typically receive steroids, antivirals, and comfort medications, all of which have since shown benefit throughout the pandemic [39]. However, it remains uncertain if these interventions directly benefit AIS. Further evaluation is necessary to identify possible benefits and effects of antivirals (i.e., remdesivir) on mitigating adverse vascular events [40], given that many articles did not investigate stroke specifically [41]. Acute ischemic stroke recurrence, defined as new ischemic lesions within one week to 90 days after a clinically symptomatic stroke, has a 2% recurrence rate, with LVOs being the most common recurrence subtype [42,43,44]. It is controversial if any of the aforementioned interventions help prevent or reduce recurrence, especially when the original presentation occurs during the same admission as the patient’s COVID-19 illness, and our study lacks data pertaining to the intensity and efficacy of certain treatment modalities (i.e., antiplatelet medications) that could play a role in prognosis [45]. Further research is necessary to identify if long COVID factors affect the incidence of recurrent AIS given the reports of persistent neurological symptoms, such as headaches and fatigue, months after COVID-19 infection [5,46]. Our study is limited in that we could not study long COVID as the 2020 NIS data does not include the 2021 ICD-10 code for this diagnosis. 

There have been concerns surrounding COVID-19 vaccination with DNA and mRNA vaccines and the potential development of complications, such as acute ischemic stroke [47,48]. In Kolahchi et al., reportedly 22 cases (51.1% of that study’s cohort) developed AIS associated with the vaccine-induced immune thrombotic thrombocytopenia (VITT; thrombocytopenia and antibodies against platelet factor 4 leading to thrombosis) four weeks after all, except one patient, received a viral vector vaccine out of > 1.3 billion (< 0.000%) COVID-19 administered vaccinations from December, 2020, to December, 2021 [47,49]. In contrast, Stamenkovic et al. found no connection between vaccination and stroke [48]. Based on these publications, the benefits outweigh the risks of vaccination (i.e., decreased severity of COVID-19 infection, hospitalization, mortality, etc.) [50]; however, supplementary literature to determine if vaccination reduces COVID-19-related AIS specifically is welcomed. 

The 2020 NIS database has provided new data that can be used to further investigate COVID-19-related AIS. Our study supports the important learning lesson that AIS with COVID leads to worse clinical outcomes and that COVID-19 infection itself can increase the risk of AIS. This information emphasizes the importance of closer inpatient monitoring and outpatient follow-up with neurology for those who develop neurologic complications due to COVID-19 illness. With this, there can be further research into the management of COVID-19-related AIS in order to improve mortality and other outcomes. This evidence also promotes and encourages vaccination.

## 5. Limitations

Our study data were obtained from the NIS, which may result in selection bias. This is a collection from a United States in-hospital database, and it lacks data in the out-patient, nursing home, and global settings. The diagnosis of acute ischemic stroke and COVID-19 were based on ICD-10 codes because NIS data lacked lab values and imaging, which may make diagnoses prone to error. Our larger sample size helps reduce the effects of these errors. The study cohorts included non-vaccinated individuals, as the FDA first approved vaccination against COVID under the EUA on 11 December 2020. It is probable that COVID-19 vaccination may alter the outcomes of acute ischemic stroke between vaccinated and unvaccinated patients.

## 6. Future Directions

The NIS 2020 database primarily includes non-vaccinated patients, as the first vaccine against COVID-19 was made available in December 2020. A recent study by Kim et al. noted that vaccination against COVID-19 is associated with reduced stroke risk in patients with COVID-19 infection [51]. Given the high mortality reported in AIS with COVID-19 infection in our study, it may serve as a valuable reference to address vaccine hesitancy in unvaccinated individuals t. In contrast, some studies have also suggested an increased risk of stroke after COVID-19 vaccination [52,53]. We could not study the effect of vaccination on AIS outcomes with COVID-19 infection. Long COVID may be associated with an increased risk of AIS stroke [54]. Conversely, patients with the COVID-19 infection and AIS may have an altered risk of different long-term outcomes from the stroke, e.g., recurrent strokes and bleeding. More studies are needed to improve understanding of stroke outcomes after a COVID-19 infection and long COVID.

Our study supports the important learning lesson that AIS with COVID leads to worse clinical outcomes and that COVID-19 infection itself can increase the risk of AIS. These results are similar to other diseases showing worse outcomes with COVID-19 infection, e.g., STEMI [51]. Successful stroke care requires complex, timely collaboration between multiple clinical teams, and factors like delay in access during the early COVID-19 pandemic may have played a role in worse outcomes [55]. A better understanding of these factors can guide the development of tools and policies to better prepare for future pandemics [55].

## 7. Conclusions

Our study found worse outcomes among patients with acute ischemic stroke with COVID-19 infection in terms of mortality and morbidity, including an increase in NIHSS scores, acute venous thromboembolism, acute myocardial infarction, cardiac arrest, septic shock, acute kidney injury, and mechanical ventilation compared with patients without COVID-19 infection. Our study primarily included non-vaccinated patients who may be at risk of worse outcomes with COVID-19 infection. These results highlight the need for more research on the role of vaccination and treatment options to improve acute ischemic stroke-related outcomes in COVID-19-positive patients.

## Figures and Tables

**Table 1:** Ischemic Stroke: patient-level characteristics for COVID-19-positive patients with acute ischemic stroke and COVID-19-negative patients with acute ischemic stroke.

Characteristics	COVID-19-Positive Patients with AIS ^1^	COVID-19-Negative Patients with AIS ^1^	*p* Value
*n* = 329,240	*n* = 6665	*n* = 322,575	
SEX (Female)	43.36%	48.33%	<0.001
Mean age years (SD)			
Male	66.18 (13.02)	67.3(13.2)	
Female	69.53 (15.25)	71.2(14.3)	
Age Groups			0.009
≥18–29	0.68%	0.63%	
30–49	10.58%	8.19%	
50–69	40.21%	39.29%	
≥70	48.54%	51.9%	
RACE			<0.001
Caucasians	51.01%	68.09%	
African American	21.83%	17.58%	
Hispanics	18.75%	8.01%	
Asian or Pacific Islander	3.6%	3.16%	
Native American	0.75%	0.41%	
Others	4.05%	2.74%	
Median Household Income			0.03
<49,999$	34.19%	29.83%	
50,000–64,999$	26.91%	27.31%	
65,000–85,999$	21.99%	23.39%	
>86,000$	16.91%	19.47%	
Insurance Status			0.02
Medicare	60.5%	64.02%	
Medicaid	12.82%	10.42%	
Private	22.27%	20.85%	
Self-pay	4.41%	4.71%	
Hospital Division			0.001
New England	3.75%	4.21%	
Middle Atlantic	14.1%	12.04%	
East North Central	16.2%	15.9%	
West North Central	7.35%	7.14%	
South Atlantic	19.05%	22.89%	
East South Central	7.8%	8.23%	
West South Central	15.45%	11.77%	
Mountain	5.4%	5.34%	
Pacific	10.88%	12.48%	
Hospital Bedsize			0.62
Small	15.75%	16.59%	
Medium	26.78%	27.35%	
Large	57.46%	56.06%	
Hospital Teaching Status			
Rural	3.3%	4.69%	
Urban non-teaching	12.83%	15.69%	
Urban teaching	83.87%	79.62%	
NIH stroke scale			<0.001
0–9	60.39%	76.24%	
10–19	22.66%	15.23%	
20–29	12.98%	7.22%	
30–39	3.98%	1.26%	
40–42	0	0.054%	
Comorbidities			
Coronary Artery Disease	22.58%	25.62%	0.01
Congestive Heart Failure	21.23%	19.69%	0.15
Hypertension	30.16%	30.5%	0.79
Diabetes Mellitus	45.99%	38.62%	<0.001
Chronic pulmonary disease	14.03%	15.76%	0.08
Chronic Kidney Disease	15.83%	16.88%	0.32
Obesity	19.05%	16.94%	0.03
Smoking	25.66%	40.84%	<0.001
Alcohol abuse	3.75%	4.95%	0.04
Cardiac arrhythmias	33.31%	33.26%	0.96

^1 ^AIS: Acute Ischemic Stroke. $: US Dollars

**Table 2:** In-hospital Outcomes for COVID-19-positive patients with acute ischemic stroke and COVID-19-negative patients with acute ischemic stroke.

Variable	COVID-19-Positive Patients with AIS ^1^	COVID-19-Negative Patients with AIS ^1^	*p* Value
In-hospital mortality (*n* =13990)	16.8% Adjusted odds ratio ^2^	3.99% 3.34 (95% CI 2.72–4.11)	<0.001
Vasopressor use	3.15% Adjusted odds ratio ^2^	1.03% 1.18 (95% CI 0.76–1.81)	0.44
Mechanical ventilation	20.48% Adjusted odds ratio ^2^	7.06% 1.90 (95% CI 1.56–2.32)	<0.001
Cardiac arrest	3.38% Adjusted odds ratio ^2^	0.65% 2.48 (95% CI 1.69–3.65)	<0.001
Septic shock	4.95% Adjusted odds ratio ^2^	0.91% 2.61 (95% CI 1.88–3.62)	<0.001
Seizure	3.9% Adjusted odds ratio ^2^	4.05% 0.75 (95% CI 0.55–1.03)	0.08
Acute VTE ^3^	7.8% Adjusted odds ratio ^2^	2.61% 2.45 (95% CI 1.98–3.04)	<0.001
Acute kidney injury	27.83% Adjusted odds ratio ^2^	15.27% 1.62 (95% CI 1.4–1.87)	<0.001
Acute myocardial infarction	8.25% Adjusted odds ratio ^2^	4.22% 1.48 (95% CI 1.18–1.85)	<0.001
AKI ^4^ requiring HD ^5^	2.1% Adjusted odds ratio ^2^	0.38% 1.81 (95% CI 1.13–2.89)	0.01
Thrombolysis	12.9% Adjusted odds ratio ^2^	12.58% 0.94 (95% CI 0.79–1.12)	0.50
Mechanical thrombectomy	8.85% Adjusted odds ratio ^2^	7.47% 0.76 (95% CI 0.61–0.95)	0.02
Mean total hospitalization charge ($)	145497 Adjusted total charge ^2^	88320 25,814$ higher	<0.001
Mean length of stay (days)	10.15 Adjusted length of stay ^2^	5.38 3.18 day higher	<0.001
Disposition			<0.001
Home/Routine	30.42%	38.14%	
SNF ^6^/LTAC ^7^/Nursing home	48.29%	40.8%	
Home health	20.31%	19.72%	
AMA ^8^	0.99%	1.35%	

^1^ AIS: Acute Ischemic Stroke. ^2^ Adjusted for age, sex, race, insurance status, discharge quarter, Elixhauser comorbidity score, teaching status and bed size, and NIH stroke scale. ^3^ VTE: Venous thromboembolism; ^4^ AKI: Acute kidney injury; ^5^ HD: Hemodialysis; ^6^ SNF: Skilled nursing facility; ^7^ LTAC: Long term acute care hospital; ^8^ AMA: Against medical advice.

**Table 3:** Mortality breakdown for COVID-19-positive patients with acute ischemic stroke and COVID-19-negative patients with acute ischemic stroke.

Variable	COVID-19 -Positive Patients with AIS ^1^	COVID-19 -Negative Patients with AIS ^1^	*p* Value
Total died (13,990)	1119	12,871	
SEX			0.003
Male	61.61%	48.6%	
Female	38.39%	51.4%	
Age Groups			0.2
≥18–29	0	0.43%	
30–49	5.8%	5.36%	
50–69	34.38%	28.4%	
≥70	59.82%	65.81%	
Race			<0.001
Caucasians	49.11%	73.31%	
African American	17.41%	11.97%	
Hispanics	21.88%	7.34%	
Asian or Pacific Islander	5.36%	3.61%	
Native American	1.34%	0.47%	
Others	4.91%	3.3%	

^1^ AIS: Acute ischemic stroke.

**Table 4:** In-hospital outcomes of propensity matched sample.

VARIABLE	COVID-19-Positive Patients with AIS ^1^	COVID-19-Negative Patients with AIS ^1^	*p* Value
In-hospital mortality (N = 13,990)	16.85% Adjusted odds ratio	4.05% 2.45 (95% CI 1.65–3.63)	<0.001
Vasopressor use	3.1% Adjusted odds ratio	1.03% 1.35 (95% CI 0.71–2.55)	0.35
Mechanical ventilation	20.19% Adjusted odds ratio	6.2% 2.08 (95% CI 1.55–2.79)	<0.001
Cardiac arrest	3.1% Adjusted odds ratio	0.4% 4.18 (95% CI 1.54–11.36)	0.005
Septic shock	4.77% Adjusted odds ratio	0.72% 3.3 (95% CI 1.48–7.36)	0.003
Seizure	3.82% Adjusted odds ratio	3.66% 0.98 (95% CI 0.61–1.55)	0.93
Acute VTE ^2^	8.11% Adjusted odds ratio	2.54% 2.55 (95% CI 1.69–3.86)	<0.001
Acute kidney injury	28.14% Adjusted odds ratio	14.31% 1.82 (95% CI 1.45–2.29)	<0.001
Acute myocardial infarction	8.51% Adjusted odds ratio	3.66% 1.72 (95% CI 1.15–2.57)	0.008
AKI ^3^ requiring HD ^4^	1.99% Adjusted odds ratio	0.48% 1.87 (95% CI 0.68–5.13)	0.22
Thrombolysis	12.64% Adjusted odds ratio	10.81% 1.12 (95% CI 0.86–1.47)	0.37
Mechanical thrombectomy	8.82% Adjusted odds ratio	6.84% 0.80 (95% CI 0.56–1.15)	0.23
Mean total hospitalization charge ($)	145,287 Adjusted total charge	84,419 31,400$ higher	<0.001
Mean length of stay (days)	10.2 Adjusted length of stay	5.67 2.8 day higher	<0.001

^1 ^AIS: Acute ischemic stroke. ^2^ VTE: Venous thromboembolism; ^3^ AKI: Acute kidney injury; ^4^ HD: Hemodialysis.

## Data Availability

Restrictions apply to the availability of these data. Data were obtained from the National Inpatient Sample database, US.

## Supplementary Materials

The following supporting information can be downloaded at: https://www.mdpi.com/article/10.3390/jcm12041340/s1, Figure S1: Flow chart of inclusion and exclusion criteria; Figure S2: Propensity matching score graph; Table S1: ICD10 Clinical Modification Codes; Table S2: Propensity Matched: Baseline Patients Characteristics.
