# Supplementary material for: COVID-19 and Acute Ischemic Stroke Mortality and Clinical Outcomes among Hospitalized Patients in the United States: Insight from National Inpatient Sample
Source: J Clin Med. 2023 Feb 8;12(4):1340. doi: 10.3390/jcm12041340 (PMC9968226; doi:10.3390/jcm12041340)

Supplementary Table S1: ICD10 Clinical Modification Codes

Variable	ICD-10 CM code
COVID-19	U071, U00, U49, U50, U85, J1282
Acute Kidney Injury	N17.XX, N99.0
Ischemic Stroke	I63.XX
Hemorrhagic stroke	I60.XX, I61.XX, G43.6XX, I97.82XX, I97.81XX, G97.31, G97.32, G97.51, G97.52, G97.61, G97.81, G97.82
Contrast allergy	Z91.041
NIHSS	R29.7XX
Cardiac arrest	I46.XX, I49.0XX, I97.12XX, I97.71XX
Smoking	F17.XX, Z87.891
Coronary Artery Disease	I25.10, I25.11, I25.118, I25.119, I252, I253, I25.4XX, I25.5, I25.6, I25.8XX, I25.7XX, I25.9XX
Acute Myocardial Infarction	I21.XX, I22.XX
Septic shock	R65.21
Seizure	G40.XX
Venous Thromboembolism	I82.4XX, I82.6XX, I82.B1X, I82.A1X, I82.C1X, I26.XX, I82.0, I82.1, I82.210, I82.220, I82.290 I82.3X, I82.890, I82.90, I82.81X
Congestive Heart Failure, Hypertension, Diabetes Mellitus, Renal failure, Chronic pulmonary disease, Obesity, cardiac arrhythmias, alcohol abuse	Elixhauser comorbidities were used
**Variable**	**ICD-10 procedure code**
Intubation	5A1945Z, 5A1955Z, 5A1935Z, 5A09357, 5A09457, 5A09557
Thrombolysis	3E03317, 3E04317, 3E05317, 3E06317, 3E08317, 3E03017, 3E04017, 3E05017, 3E06017, 3E08017
Mechanical Thrombectomy	03CG3Z6, 03CG3Z7, 03CG3ZZ, 03CG4Z6, 03CG4ZZ, 03CK3Z7, 03CK3ZZ, 03CK4ZZ, 03CL3Z7, 03CL3ZZ, 03CL4ZZ
Vasopressor use	3E030XZ, 3E033XZ, 3E040XZ, 3E043XZ, 3E050XZ, 3E053XZ, 3E060XZ, 3E063XZ
Hemodialysis	5A1D70Z, 5A1D90Z, 5A1D80Z, 5A1D00Z, 5A1D60Z

Supplementary Table S2: Propensity Matched: Baseline Patients Characteristics

1:1 Propensity matched variables: Age, race, income and insurance status, sex

CHARACTERISTICS	COVID-19 positive patients with stroke	COVID negative patients with stroke	P value
N=12580	N=6290	N=6290	
Mean age years (SD)	68.01 (9.7)	68.28 (9.5)	0.64
Sex (Female)	44.52%	44.59%	0.96
AGE GROUPS			0.80
≥18-29	0.72%	0.64%	
30-49	10.17%	9.14%	
50-69	39.19%	40.38%	
≥70	49.92%	49.84%	
RACE			0.99
Caucasians	51.59%	51.51%	
African American	21.78%	22.18%	
Hispanics	18.44%	18.52%	
Asian or Pacific Islander	3.74%	3.74%	
Native American	0.79%	0.87%	
Others	3.66%	3.18%	
MEDIAN HOUSEHOLD INCOME			0.99
<49,999$	34.34%	34.5%	
50,000 – 64,999$	27.03%	26.95%	
65,000 – 85,999$	21.86%	21.94%	
>86,000$	16.77%	16.61%	
INSURANCE STATUS			0.74
Medicare	60.33%	60.65%	
Medicaid	12.88%	11.45%	
Private	22.42%	23.29%	
Self-pay	4.37%	4.61%	

$: US Dollars

Supplemental Figure S1: Flow chart of inclusion and exclusion criteria

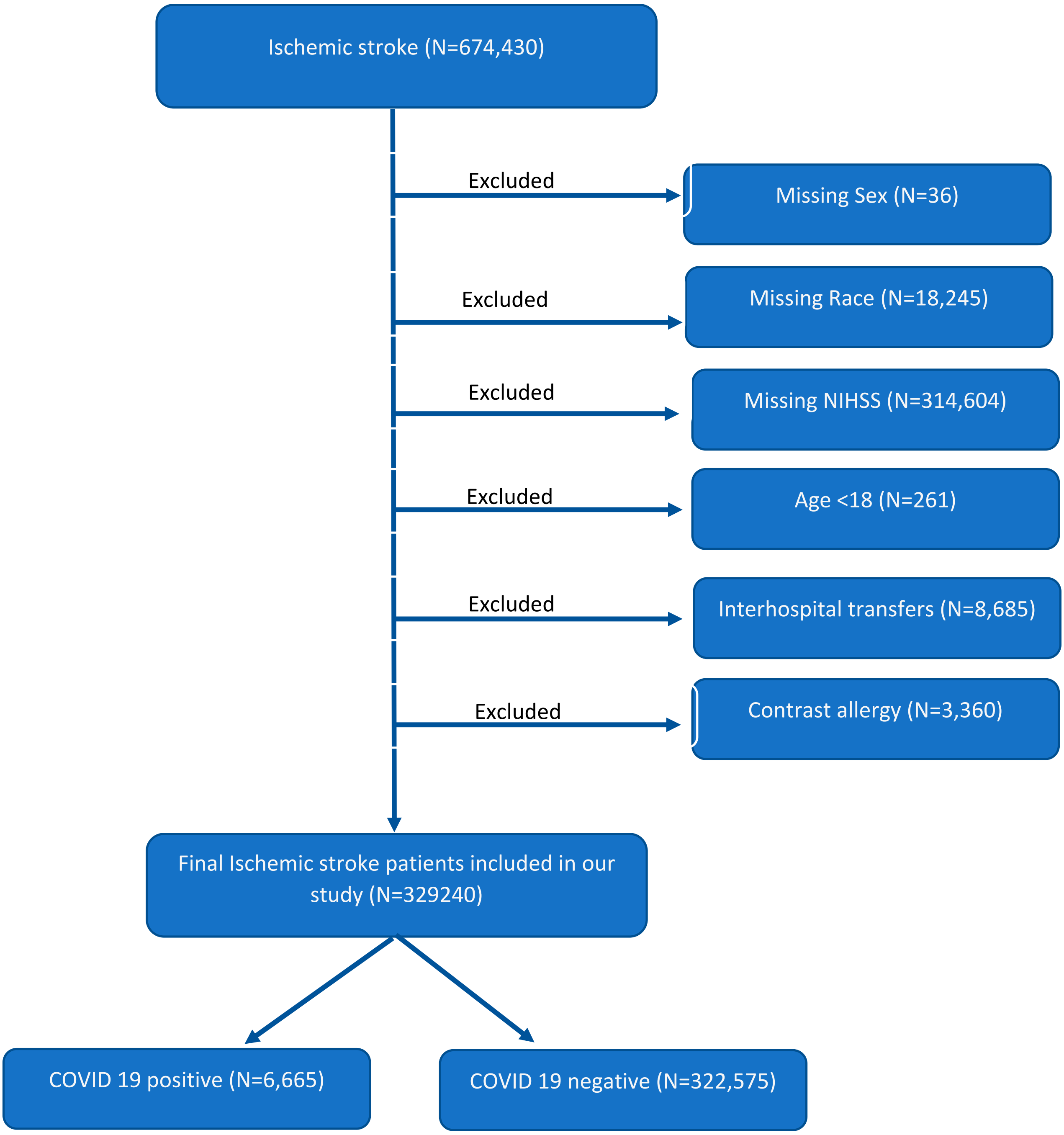

Supplemental Figure S2: Propensity matching score graph

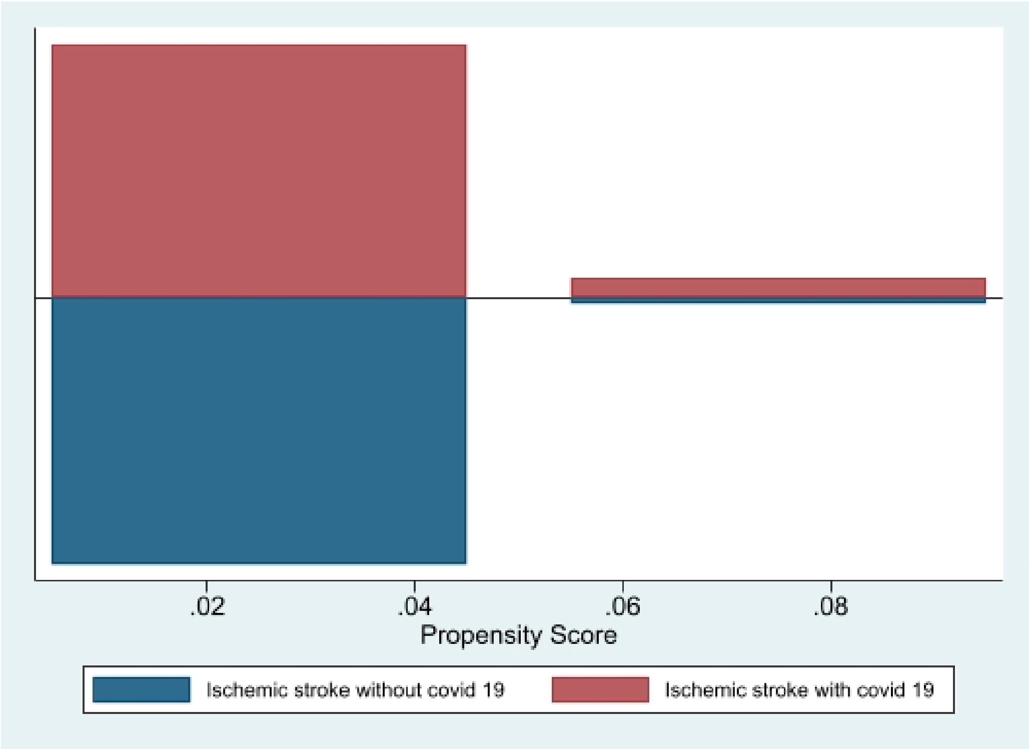

Supplement: Supplementary file 1 [file jcm-12-01340-s001.zip › jcm-2203665-supplementary.pdf]
